# miR-600 promotes ovarian cancer cells stemness, proliferation and metastasis via targeting KLF9

**DOI:** 10.1186/s13048-022-00981-7

**Published:** 2022-05-03

**Authors:** Lili Shan, Pingping Song, Yangyang Zhao, Na An, Yanqiu Xia, Yue Qi, Hongyan Zhao, Jing Ge

**Affiliations:** 1 Department of Gynaecology and Obstetrics, the Veterans General Hospital of Liaoning Province, the Second Affiliated Hospital of Shenyang Medical College, Shenyang, 110002 Liaoning China; 2 Department of Endoscopy, Northern Theater General Hospital, Shenyang, 110011 Liaoning China; 3 Department of Neonatology, Northern Theater General Hospital, Shenyang, 110011 Liaoning China

**Keywords:** Ovarian cancer, miR-600, KLF9, Stemness, Metastasis

## Abstract

Previous studies have revealed that miRNAs participate in the pathogenesis of ovarian cancer; however, whether miR-600 is also involved remains unclear. In this study, we aimed to investigated the role of miR-600 in ovarian cancer progression. Here, miR-600 expression was significantly upregulated in ovarian cancer tissues and stem cells. Functional studies showed that miR-600 promoted ovarian cancer cell stemness, proliferation and metastasis. Mechanistic studies revealed that Kruppel like factor 9 (KLF9) was indicated as the target of miR-600. The luciferase reporter assay suggested that miR-600 directly bound to the 3′-untranslated region of KLF9. Additionally, miR-600 expression was negatively associated with KLF9 expression in human ovarian cancer tissues. Si-KLF9 partially abolished the discrepancy of self-renewal, growth and metastasis capacity between miR-600 knockdown ovarian cancer cells and control cells. In conclusion, our results suggest that miR-600 promotes ovarian cancer cell stemness, proliferation and metastasis via directly downregulating KLF9, and impairing miR-600 levels may be a new treatment strategy for ovarian cancer in the future.

**Supplementary Information:**

The online version contains supplementary material available at 10.1186/s13048-022-00981-7.

## Introduction

Ovarian cancer (OC) is a high-fatality gynecological malignancy with a five-year survival rate of less than 50% after diagnosis in the world [1]. Most ovarian cancer patients are diagnosed at advanced stage due to an asymptomatic and highly differentiated nature [2]. For advanced ovarian cancer patients, it’s hard to be thoroughly resected and the rate of postoperative recurrence and metastasis is very high [3]. Although chemotherapy and immunotherapy are used for advanced ovarian cancer patients who cannot be resected, the results are still unsatisfactory [4, 5]. Many ovarian cancer patients develop resistance after a period of treatment [6, 7]. Therefore, it’s urgent to explore the underlying mechanism of tumorigenesis and search for new therapeutic targets.

MicroRNAs (miRNAs), non-coding short-chain (19–22 nt) RNAs, modulate downstream genes expression through transcriptional and/or post-transcriptional regulation [8, 9]. Several recent studies have indicated that dysregulated miRNAs expression is closely correlated with tumorigenesis, proliferation, metastasis and poor prognosis for many human cancers [10–14]. miR-600 is a newly discovered miRNA, and its role and mechanism in biological development and disease are not completely understood. Previous studies reported that miR-600 acts as tumor suppressor and a diagnostic and prognostic biomarker in breast cancer patients [15]. Another study reported that miR-600 inhibits lung cancer via downregulating the expression of METTL3 [16]. These studies suggest that dysregulated miR-600 plays an important role in promoting cancer progression. However, whether miR-600 contributes to ovarian cancer progression remains unknown, and the molecular mechanism requires further investigation.

In this study, we present the first evidence that miR-600 was upregulated in ovarian cancer tissues and stem cells. miR-600 promoted ovarian cancer cell stemness, proliferation and metastasis via downregulating KLF9. Our results highlighted the importance of miR-600 in regulating the progression of ovarian cancer and could be a potential therapeutical target.

## Materials and methods

### Collection of clinical tissue specimens

Thirty-four ovarian cancer tissues and adjacent normal tissues; eighteen metastatic foci and matched primary ovarian cancer tissues; eighteen recurrence ovarian cancer tissues and matched primary ovarian cancer tissues were all obtained from ovarian cancer patients under surgery at the First Affiliated Hospital of China Medical University (Shenyang, Liaoning, China). Written informed consent about tissue donation for study purposes was obtained from all the participants before the surgery. The study design was reviewed and approved by the Clinical Research Ethics Committees of the First Affiliated Hospital of China Medical University, and all experimental methods were carried out in accordance with the guidelines of the Declaration of Helsinki.

### Cell lines and cell culture

Human ovarian carcinoma cell lines HO8910 and A2780 were obtained from the American Type Culture Collection (Manassas, VA). All cells were maintained in RPMI-1640 medium (Solarbio, Beijing, China) containing 10% fetal bovine serum (Thermo Fisher, Wilmington, DE, USA) and 1% penicillin-streptomycin solution (Procell, Wuhan, China), and cultured at 37 °C in 5% CO_2_.

HO8910 and A2780 cells were dissociated with 0.5% trypsin and seeded into six-well plates. HO8910 and A2780 cells were infected with miR-600 knockdown virus and control virus. HO8910 and A2780 cells were infected with si-KLF9 and control siRNA. Then the stable infectants were screening by using puromycin as before [17]. miR-600 knockdown virus virus was obtained from Shanghai GenePharma (Shanghai, China). KLF9 siRNA was also obtained from Shanghai GenePharma (Shanghai, China).

#### Spheroids formation assay

miR-600 knockdown ovarian cancer cells and control cells were seeded in a 96-well ultra-low attachment culture plate for 7 days (300/well), and the total number of spheres was counted under the microscope.

#### In vitro limiting dilution assay

Various numbers of miR-600 knockdown ovarian cancer cells and control cells (2, 4, 8, 16, 32, 64/well) were seeded into 96-well ultra-low attachment culture plates for one week. CSC proportions were analyzed using Poisson distribution statistics and the L-Calc Version 1.1 software program (Stem Cell Technologies, Inc., Vancouver, Canada) as described [18].

### Cell proliferation assays

For CCK8 assay, miR-600 knockdown ovarian cancer cells and control cells were seeded in 96-well plates (3 × 10^3^ cells per well). ATP activity was measured using a Cell Counting Kit-8 at indicated time points (0, 24, 48, 72, 96 h). The procedure was as follows: The cell suspension (100 μl/well) was inoculated in a 96-well plate, and the plate was pre-incubated in a humidified incubator at 37 °C for 1 h. This was followed by the addition of 10 μl of the CCK-8 solution to each well of the plate, and incubation of the plate for 1 h in the incubator. Finally, the absorbance was measured at 450 nm using a microplate reader (Synergy H1; BioTek Instruments, Inc., Winooski, VT, USA).

For colony formation assay, miR-600 knockdown ovarian cancer cells and control cells were cultured in 12-well plates (3 × 10^3^ cells/well). The cells were incubated at 37 °C for 7 days and then fixed with 10% neutral formalin for more than 4 h. The cells were dyed with crystal violet (Beyotime, Haimen, China). The cells were photographed under a microscope (Olympus, Tokyo, Japan).

For cell EdU immunofluorescence staining, miR-600 knockdown ovarian cancer cells and control cells were seeded into 96-well plates (3 × 10^3^ cells per well) and performed using the EdU Kit (RiboBio) at 48 h. The results were quantified with a Zeiss axiophot photomicroscope (Carl Zeiss) and Image-Pro plus 6.0 software.

### Cell migration assays

For cell migration experiments, 2 × 10^5^ miR-600 knockdown ovarian cancer cells and control cells were seeded into the upper chamber of a polycarbonate transwell in serum-free RPMI-1640 medium. The lower chamber was added with RPMI-1640 medium containing 20% FBS as chemoattractant. The cells were incubating for 24 h and the chamber was fixed with 10% neutral formalin for more than 4 h. The cells were dyed with crystal violet (Beyotime). The cells were then counted under a microscope (Olympus) and the cell number is expressed as the average number of the cells in each field.

### Cell invasion assays

For cell invasion experiments, 2 × 10^5^ miR-600 knockdown ovarian cancer cells and control cells were seeded into the upper chamber of a polycarbonate transwell in serum-free RPMI-1640 medium. The lower chamber was added with RPMI-1640 medium containing 20% FBS as chemoattractant. The cells were incubating for 36 h and the chamber was fixed with 10% neutral formalin for more than 4 h. The cells were dyed with crystal violet (Beyotime). The cells were then counted under a microscope (Olympus) and the cell number is expressed as the average number of the cells in each field.

### Animal models

For in vivo limiting dilution assay, different concentrations of HO8910 miR-600 sponge or control cells (1 × 10^3^, 5 × 10^3^, 1 × 10^4^, 5 × 10^4^) were mixed with Matrigel gel (1:1) and then injected subcutaneously into NOD-SCID mice. Mice were sacrificed seven weeks post inoculation and tumors incidence was examined.

For xenograft formation assay, HO8910 miR-600 sponge or control cells (2 × 10^6^) were injected subcutaneously into nude mice. Mice were sacrificed seven weeks post inoculation and tumors were collected and examined.

For lung metastasis model, HO8910 miR-600 sponge or control cells (2 × 10^6^) were injected into the tail vein of nude mice. Mice were killed 12 weeks after inoculation and consecutive sections of the whole lung were subjected to haematoxylin-eosin staining. All the metastatic lesions in lung were calculated microscopically to evaluate the development of pulmonary metastasis.

### Luciferase reporter assays

The cDNA fragment of KLF9 3′-UTR that contained the wild-type or mutant miR-600 binding site was inserted into the miRNA reporter vector (Promega, Madison, WI). Briefly, miR-600 knockdown ovarian cancer cells and control cells were transfected with pMIR-reporter luciferase vector containing a specific sequence of wild-type or mutant KLF9 fragment, using siRNA transfection (Invitrogen, NY, USA). Cells were collected and lysed for luciferase detection 48 h after transfection. The relative luciferase activity was normalized against to the Renilla luciferase activity [19].

### Real-time PCR

Total RNA from cells or tissues was extracted using the TRIzol reagent (Takara) according to the manufacturer’s instructions. Reverse transcription reactions for miRNAs were performed with SYBR PrimeScriptTM miRNA RT-PCR Kit (TaKaRa Bio Group, Shiga, Japan). U6 RNA was used as the internal control. All samples were normalized to the internal controls, and fold changes were calculated via the relative quantification method (2^-ΔΔCT^).

The total cells RNA was extracted by using Trizol reagent (Invitrogen, 15,596–018). Total cDNAs were synthesized by ThermoScript TM RT-PCR system (Invitrogen, 11,146–057). The total mRNA amount presented in the cells was measured by RT-PCR using the ABI PRISM 7300 sequence detector (Applied Biosystems). The KLF9 primer sequences were forward: 5′ AGATGTGTCCCAAAGCTCCG 3′, reverse: 5′ TACCCCTACAGCCTCGAACA 3′. The β-actin was used as reference for relative expression calculation and its primer sequences were forward: 5′ GGCCCAGAATGCAGTTCGCCTT 3′, reverse: 5′ AATGGCACCCTGCTCACGCA 3′.

### Western blotting assays

The western blotting analysis was used to detect protein KLF9 and β-actin expression. RIPA cell lysate containing protease inhibitor was used to lyse the cells for 30 min. After centrifugation at 12,000 g for 15 min at 4 °C, the supernatant was obtained. After electrophoresis, the protein sample was transferred to a PVDF membrane, and the 5% skim milk powder was sealed at room temperature for 1 h. Rabbit antihuman KLF9 antibody (Proteintech, Chicago, USA), PARP antibody (Proteintech, Chicago, USA) or mouse anti-human GAPDH antibody (Proteintech, Chicago, USA) were added separately and incubated overnight at 4 °C. After washing with TBST, membranes were incubated with secondary antibody at room temperature for 2 h. TBST was used to clean the membrane 3 times. Bands were detected using the ECL Kit and β-actin was used as a loading control.

### Statistical analysis

All statistical analyses were performed using GraphPad Prism (GraphPad Software, Inc. La Jolla, USA). Statistical analysis was carried out using t test or Bonferroni Multiple Comparisons Test: *p < 0.05. A *p* value of less than 0.05 was considered statistically significant.

## Results

### miR-600 expression was upregulated in ovarian cancer tissues and stem cells

**Fig. 1:**
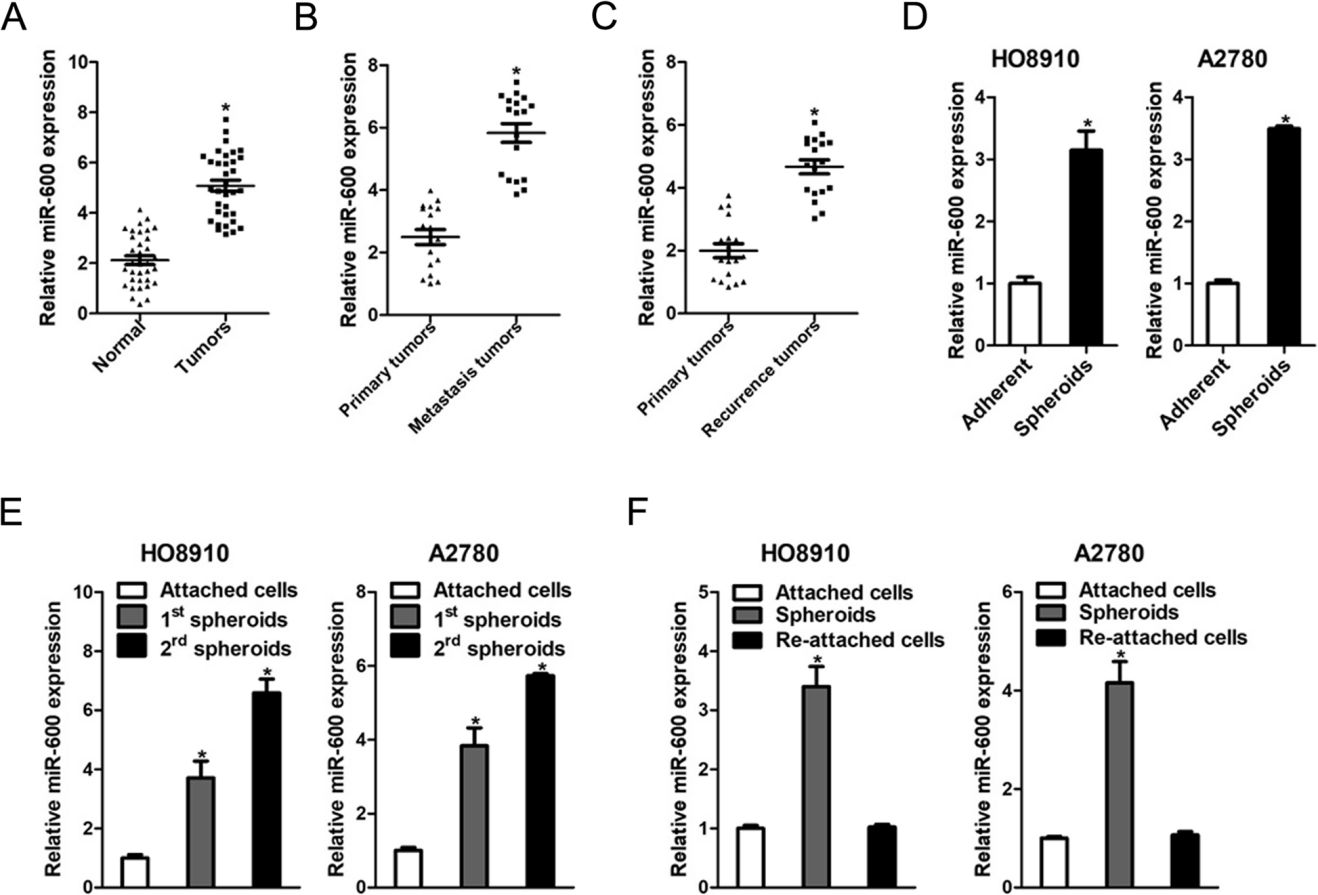
miR-600 is upregulated in ovarian cancer tissues and stem cells. **A**. The expression of miR-600 in ovarian cancer tissues and corresponding normal tissues was checked by real-time PCR assay (*n* = 34). **B**. The expression of miR-600 in paired primary ovarian cancer tissues and metastatic ovarian cancer tissues was determined by real-time PCR assay (*n* = 18). **C**. The expression of miR-600 in paired primary ovarian cancer tissues and recurrent ovarian cancer tissues was examined by real-time PCR assay (*n* = 18). **D**. The expression of miR-600 in ovarian cancer spheres and adherent cells was checked by real-time PCR assay. **E**. Real-time PCR analysis of miR-600 expression in in serial passages of ovarian cancer spheroids. **F**. Real-time PCR analysis of miR-600 expression in ovarian cancer adherent cells, spheres and re-adherent cells

To explore the expression characteristics of miR-600 in ovarian cancer, we measured the expression of miR-600 in 34 ovarian cancer tissues and adjacent normal tissues using real-time PCR. miR-600 expression was increased in ovarian cancer tissues compared with adjacent tissues (Fig. 1A). Moreover, we observed that miR-600 expression significantly elevated in metastasis ovarian cancer tissues compared with the matched primary ovarian cancer tissues (Fig. 1B). In addition, increased miR-600 expression was found in recurrent ovarian cancer tissues compared with the matched primary ovarian cancer tissues (Fig. 1C).

Next, we enriched cancer stem cells by spheroids formation. As shown in Fig. 1D, miR-600 expression was increased in ovarian cancer spheres derived from ovarian cancer cells. In serial passages of ovarian cancer spheroids, miR-600 expression gradually increased (Fig. 1E). Notably, miR-600 level was recovered to origin level when the spheres were reattached (Fig. 1F).

### Knockdown of miR-600 inhibited ovarian cancer stem cells expansion

**Fig. 2:**
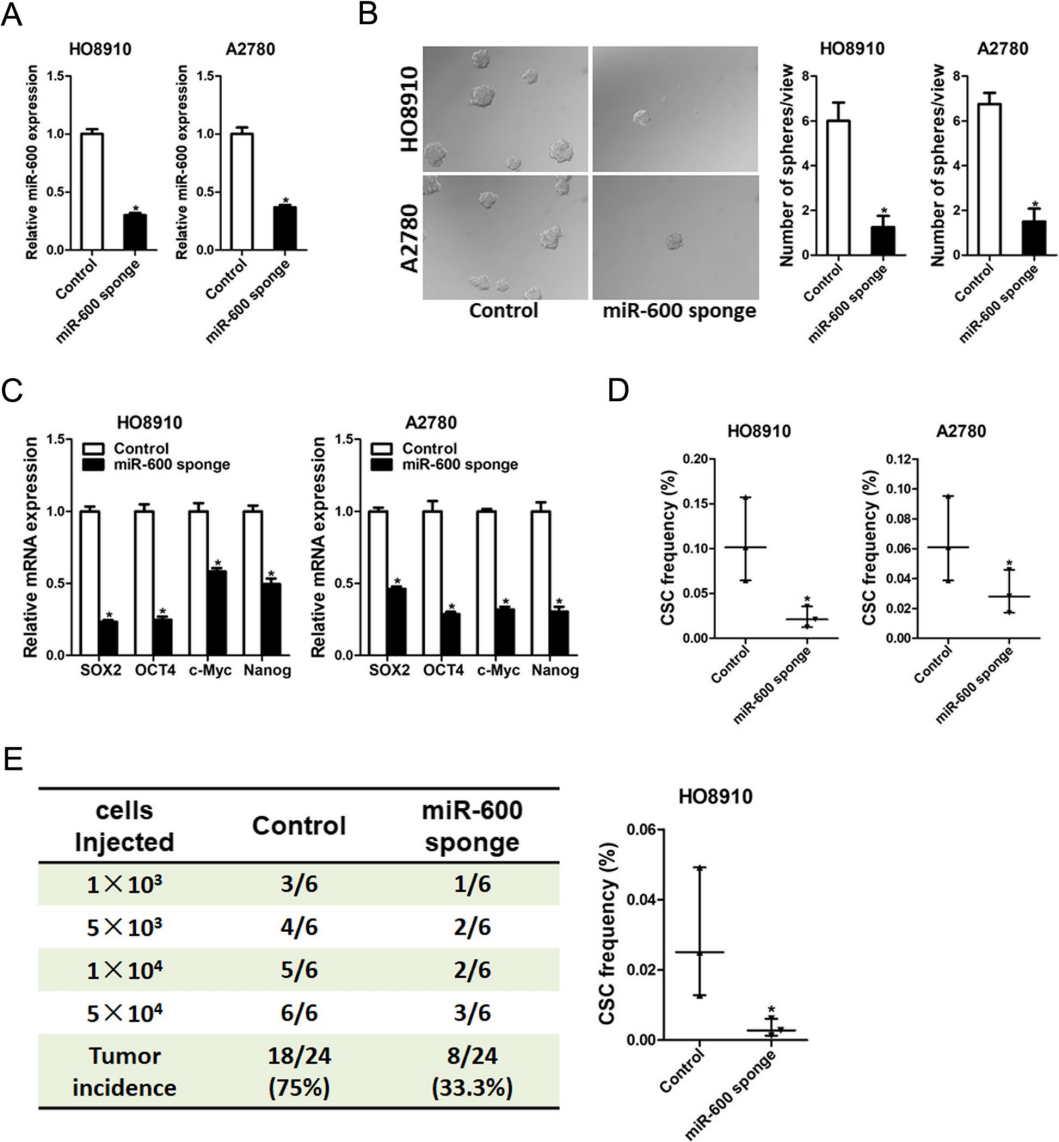
miR-600 knockdown inhibits ovarian cancer stem cells expansion. **A**. The knockdown effect of miR-600 in HO8910 and A2780 cells was checked by real-time PCR analysis. **B**. Representative images of ovarian cancer spheroids generated from miR-600 knockdown ovarian cancer cells and control cells. The number of spheroids was counted and compared. **C**. The expression of SOX2, OCT4, c-Myc and Nanog in miR-600 knockdown ovarian cancer cells and control cells was determined by real-time PCR assay. **D**. The frequency of ovarian cancer stem cells in miR-600 knockdown ovarian cancer cells and control cells was compared by in vitro limiting dilution assay. **E**. miR-600 knockdown ovarian cancer cells and control cells were inoculated into NOD-SCID mice subcutaneously, and the tumorigenicity was evaluated two months post inoculation. The frequency of CSCs was calculated

To explore the potential role of miR-600 in regulating the malignant biological behaviors of ovarian cancer cells, the miR-600 knockdown and overexpression ovarian cancer cells were used (Fig. 2A and supplementary Fig. 1A). As expected, spheroid formation ability was impaired in miR-600 knockdown ovarian cancer cells and enhanced in miR-600 overexpression ovarian cancer cells (Fig. 2B and supplementary Fig. 1B). Consistently, miR-600 knockdown inhibited the levels of stemness-like markers such as SOX2, OCT4, c-Myc and Nanog (Fig. 2C). Furthermore, in vitro limited dilution assays showed that miR-600 knockdown decreased the proportion of cancer stem cells (CSCs) in ovarian cancer cells (Fig. 2D). Conversely, miR-600 overexpression increased the proportion of CSCs in ovarian cancer cells (supplementary Fig. 1C). To further determine the effect of miR-600 on the tumorigenicity of ovarian cancer stem cells, miR-600 knockdown and control cells were inoculated into NOD/SCID mice. In vivo limiting dilution assay revealed that miR-600 knockdown significantly reduced tumor incidence and CSC frequency (Fig. 2E).

### miR-600 facilitated ovarian cancer cells proliferation and metastasis

**Fig. 3:**
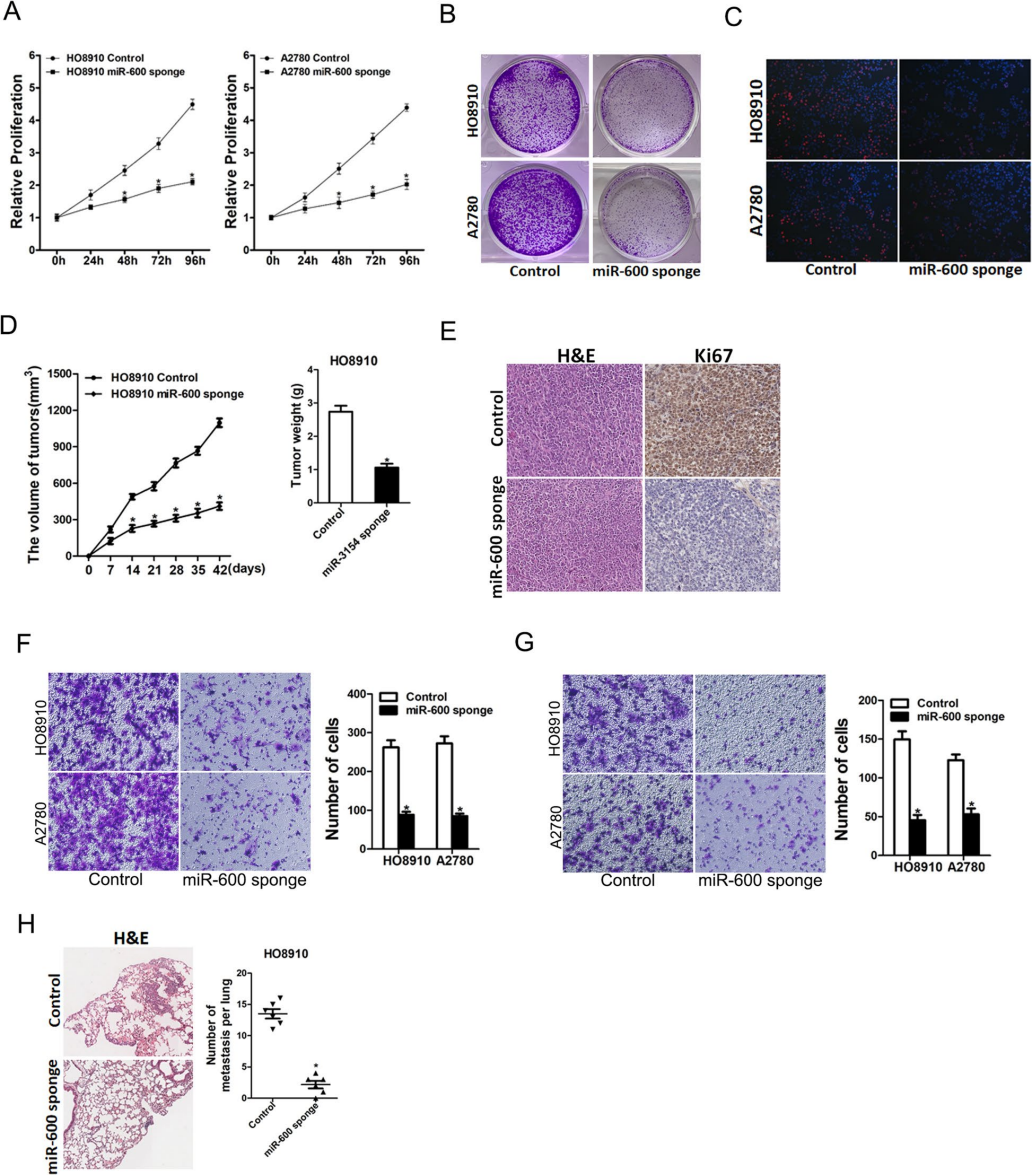
miR-600 knockdown suppresses ovarian cancer cells proliferation and metastasis. **A**. The proliferation curves of miR-600 knockdown ovarian cancer cells and control cells was measured by using CCK-8 assays. **B**. Colony formation assays of miR-600 knockdown ovarian cancer cells and control cells. **C**. The proliferation of miR-600 knockdown ovarian cancer cells and control cells were performed by EdU immunofluorescence staining assay. **D**. HO8910 miR-600 sponge or control cells (2 × 10^6^) were subcutaneously injected into nude mice (*n* = 6) for xenograft assay. Tumor growth curve and average weight in each group was shown. **E**. Representative images of H&E staining of HO8910 miR-600 sponge or control cells formed xenografted tumors. **F**. The migration ability of miR-600 knockdown ovarian cancer cells and control cells were performed utilizing polycarbonate membrane inserts in a 24-well plate. **G**. The invasive ability of miR-600 knockdown ovarian cancer cells and control cells was analyzed using Matrigel-coated Boyden chamber. **H**. HO8910 miR-600 sponge and its control cells were inoculated via tail vein for 12 weeks. The number of lung metastatic foci in each group (*n* = 6) were also calculated

In further study, CCK8 assay demonstrated that miR-600 knockdown inhibited cell growth in ovarian cancer cells (Fig. 3A). Conversely, miR-600 overexpression promoted cell growth in ovarian cancer cells (supplementary Fig. 2A). Next, we found that miR-600 knockdown ovarian cancer cells formed less and smaller colonies (Fig. 3B). 5-ethynyl-2′-deoxyuridine (EdU) staining showed that ovarian cancer cells proliferation was suppressed by miR-600 knockdown a and enhanced by miR-600 overexpression (Fig. 3C and supplementary Fig. 2B). More importantly, HO8910 miR-600 knockdown cells and control cells were inoculated into node-mice. We found that miR-600 knockdown ovarian cancer cells exhibited attenuated xenografted tumor growth, tumor size and tumor weight in vivo (Fig. 3D), which suggesting that interference of miR-600 inhibited ovarian cancer growth.

Next, we also explore the potential role of miR-600 in ovarian cancer cells metastasis. As expected, transwell assay demonstrated that miR-600 knockdown attenuated the migration ability of ovarian cancer cells (Fig. 3E). Matrigel invasion chamber assay revealed that the invasion ability was impaired in miR-600 knockdown ovarian cancer cells (Fig. 3F). More importantly, nude mice inoculated with HO8910 miR-600 sponge cells via tail vein displayed fewer micrometastatic lesions in lung as compared with control cells (Fig. 3G). Taken together, our results showed that miR-600 promoted ovarian cancer cells metastasis.

### KLF9 was the directly target of miR-600

**Fig. 4:**
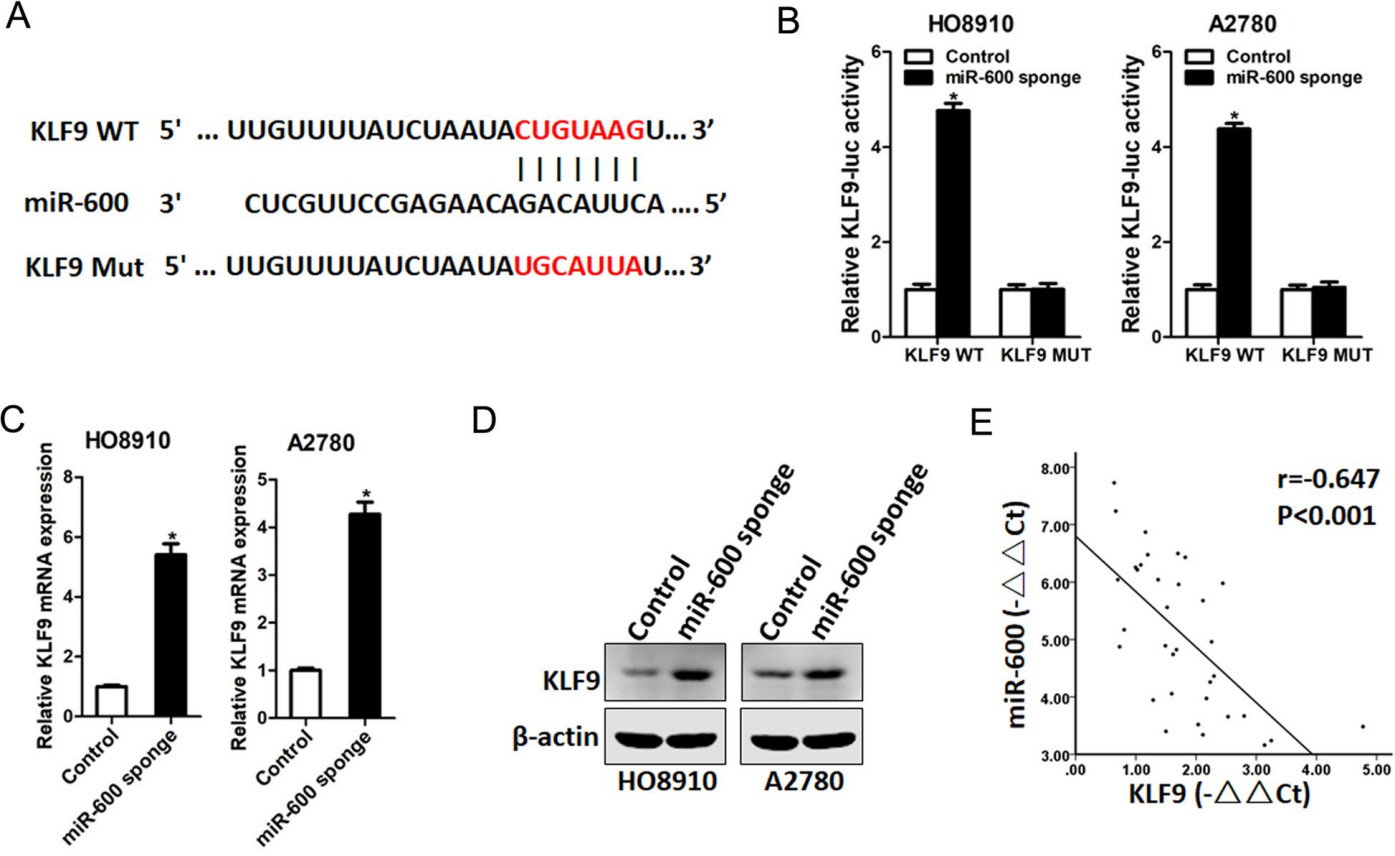
KLF9 is the directly target of miR-600 in ovarian cancer cells. **A**. A potential target site for miR-600 in the 3′-UTR of human KLF9 mRNA, as predicted by the program Targetscan and miRBase. To disrupt the interaction between miR-600 and KLF9 mRNA, the target site was mutated. **B**. Luciferase reporter assays of miR-600 knockdown ovarian cancer cells and control cells transfected with wild-type or mutant KLF9 3′-UTR constructs. **C**. Real-time PCR analysis KLF9 mRNA expression in miR-600 knockdown ovarian cancer cells and control cells. **D**. Western blot analysis of KLF9 protein expression in miR-600 knockdown ovarian cancer cells and control cells. GAPDH was used as the loading control. **E**. Significant correlation was observed between miR-600 and KLF9 expression in human ovarian cancer tissues (*n* = 34)

To investigate the downstream regulatory mechanism of miR-600 in ovarian cancer cells, we searched the TargetScan database and identified candidate KLF9. To confirm binding between miR-600 and the KLF9 3′-UTR. We performed assays with a wild-type or mutated KLF9 3′-UTR-coupled luciferase reporter (Fig. 4A). The results of the luciferase reporter assay indicated that miR-600 knockdown significantly increased luciferase activity compared with the negative control, while impaired through mutation of miR-600 binding sites within the KLF9 3′-UTR region (Fig. 4B). These results suggested that miR-600 directly bound to the 3′-UTR of KLF9. Real-time PCR and western blot further revealed that KLF9 mRNA and protein levels was increased in miR-600 knockdown ovarian cancer cells (Fig. 4C&D). Consistent with this, levels of KLF9 and miR-600 expression were negatively correlated in human ovarian cancer tissues (Fig. 4E).

### miR-600 promoted ovarian cancer cells progression via inhibiting KLF9

**Fig. 5:**
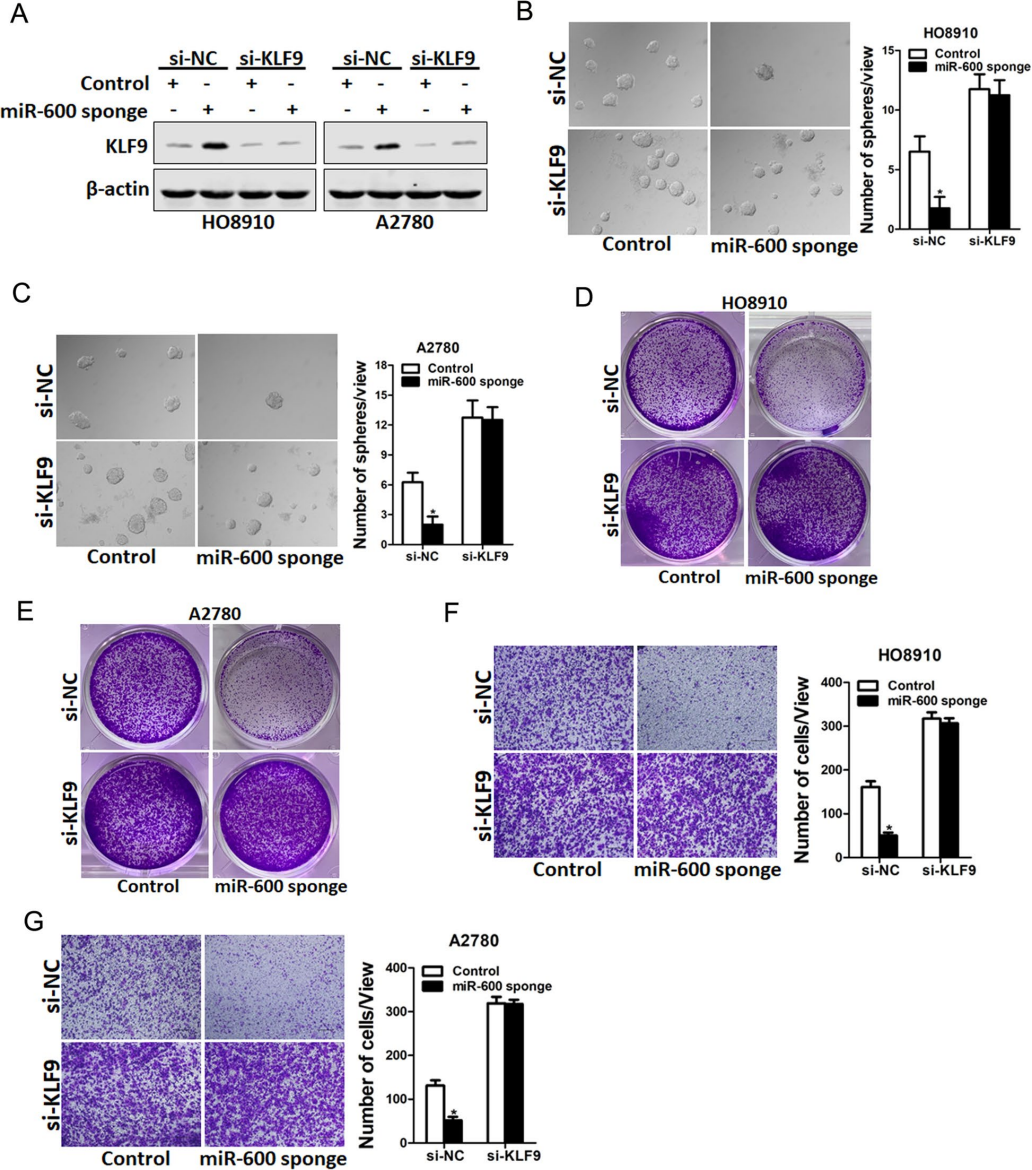
miR-600 facilitates ovarian cancer cells progression via inhibiting KLF9. **A**. HO8910 miR-600 sponge or A2780 miR-600 sponge and their control cells were transfected with si-KLF9 or control siRNA and then subjected to western blot assay. **B&C**. miR-600 knockdown ovarian cancer cells and control cells were transfected with si-KLF9 or control siRNA and then subjected to spheroids formation assay. **D&E**. miR-600 knockdown ovarian cancer cells and control cells were transfected with si-KLF9 or control siRNA and then subjected to colony formation assay. **F&G**. miR-600 knockdown ovarian cancer cells and control cells were transfected with si-KLF9 or control siRNA and then subjected to Invasion assay

Next, we investigated the role of KLF9 in miR-600-mediated ovarian cancer cells progression. As siRNA of KLF9 efficiently decreased the expression of KLF9 (Fig. 5A), we then detected the effect of siRNA of KLF9 on the progression of ovarian cancer cells. As expected, si-KLF9 recovered the spheroids formation ability of miR-600 knockdown ovarian cancer cells (Fig. 5B&C). Moreover, the proliferation and invasive capacity of miR-600 knockdown ovarian cancer cells could be restored through interference of KLF9 (Fig. 5D-G). Taken together, the above results demonstrated that KLF9 was required for miR-600-mediated ovarian cancer cells progression.

## Discussion

Ovarian cancer is one the most common primary gynecological malignancy and causes cancer-related deaths for women [20]. Understanding the mechanisms responsible for ovarian cancer tumorigenesis and progression is therefore critical for finding new therapeutic targets for this deadly disease. In this study, for the first time, we clarify that miR-600 was upregulated in ovarian cancer tissues and stem cells that determines the stemness, proliferation and metastasis of ovarian cancer.

The roles of miRNAs in ovarian cancer progression have been extensively explored. For instance, miR-522 has been identified to promotes ovarian cancer progression by regulating PTEN pathway [21]. miR-4461 has been revealed to promotes the proliferation and metastasis of ovarian cancer cells and cisplatin resistance via targeting PTEN [22]. miR-600 is a newly discovered miRNA, and its role and mechanism in biological development and disease are not completely understood. In the present study, miR-600 was revealed to be upregulated in ovarian cancer tissues and stem cells. CSCs are self-renewing cells that can generate heterogeneous tumor cells [23, 24]. Several recent studies have indicated that cancer stem cells play an important role in tumor survival, proliferation, metastasis and recurrence [25, 26]. In the current study, we confirmed that promoted ovarian cancer cells self-renewal and tumorigenesis. Additionally, we also found that promoting of miR-600 enhanced the proliferation and metastasis ability of ovarian cancer cells in vitro and in vivo, indicating that miR-600 might be involved in tumor progression. Together, our data indicate that miR-600 has an important function in ovarian cancer progression, which also indicate that miR-600 is a potential therapeutic target.

KLF9 belongs to the KLF transcriptional factor family and has been reported to involved in the regulation of diverse biological processes [27]. Several recent studies have indicated that KLF9 is downregulated in various types of human cancers and associated with cancers tumorigenicity, proliferation and metastasis [28–30]. In addition, KLF9 was reported to be involved in the regulation of CSCs [31, 32]. In this study, through bioinformatics analysis in the Targetscan database, we discovered KLF9 was a downstream target gene of miR-600. miR-600 regulated KLF9 expression and may therefore promote the progression of ovarian cancer. Considering the important role of miR-600/KLF9 axis in ovarian cancer cells stemness, proliferation and metastasis, we believe that targeting the miR-600/KLF9 axis could be a novel therapeutic strategy for ovarian cancer.

In conclusion, we for first revealed that miR-600 was upregulated in ovarian cancer tissues and stem cells. miR-600 promoted ovarian cancer cells stemness, proliferation and metastasis via downregulating KLF9. These findings of the present study not only shed a new light on the mechanism of ovarian cancer but suggest a potential therapeutic target against ovarian cancer patients.

## Supplementary Information

**Additional file 1**

## Data Availability

Data generated from the study are available from the corresponding author on reasonable request.
