# Supplementary material for: miR-600 promotes ovarian cancer cells stemness, proliferation and metastasis via targeting KLF9
Source: J Ovarian Res. 2022 May 3;15:52. doi: 10.1186/s13048-022-00981-7 (PMC9063371; doi:10.1186/s13048-022-00981-7)
Supplement: Supplementary file 1 — Additional file 1 [file 13048_2022_981_MOESM1_ESM.docx]

**miR-600 promotes** **ovarian cancer cells stemness, proliferation and metastasis via targeting KLF9**

Lili Shan^1,#^, Pingping Song^1,#^, Yangyang Zhao^1,#^, Na An^2^, Yanqiu Xia^3^, Yue Qi^1^, Hongyan Zhao^2^, Jing Ge^1,*^

^1^Department of Gynaecology and Obstetrics, the Veterans General Hospital of Liaoning Province, the Second Affiliated Hospital of Shenyang Medical College, 110002, Liaoning, China.

^2^Department of Endoscopy, Northern Theater General Hospital,110011, Liaoning, China.

^3^Department of Neonatology, Northern Theater General Hospital,110011, Liaoning, China.

^#^These authors contribute equally to this work.

**Corresponding authors:** Dr. Jing Ge, Department of Obstetrics and Gynecology, the Veterans General Hospital of Liaoning Province, the Second Affiliated Hospital of Shenyang Medical College, 110002, Liaoning, China. Email address: jingge1031@163.com.

**Running title:** miR-600 promotes ovarian cancer cells progression.


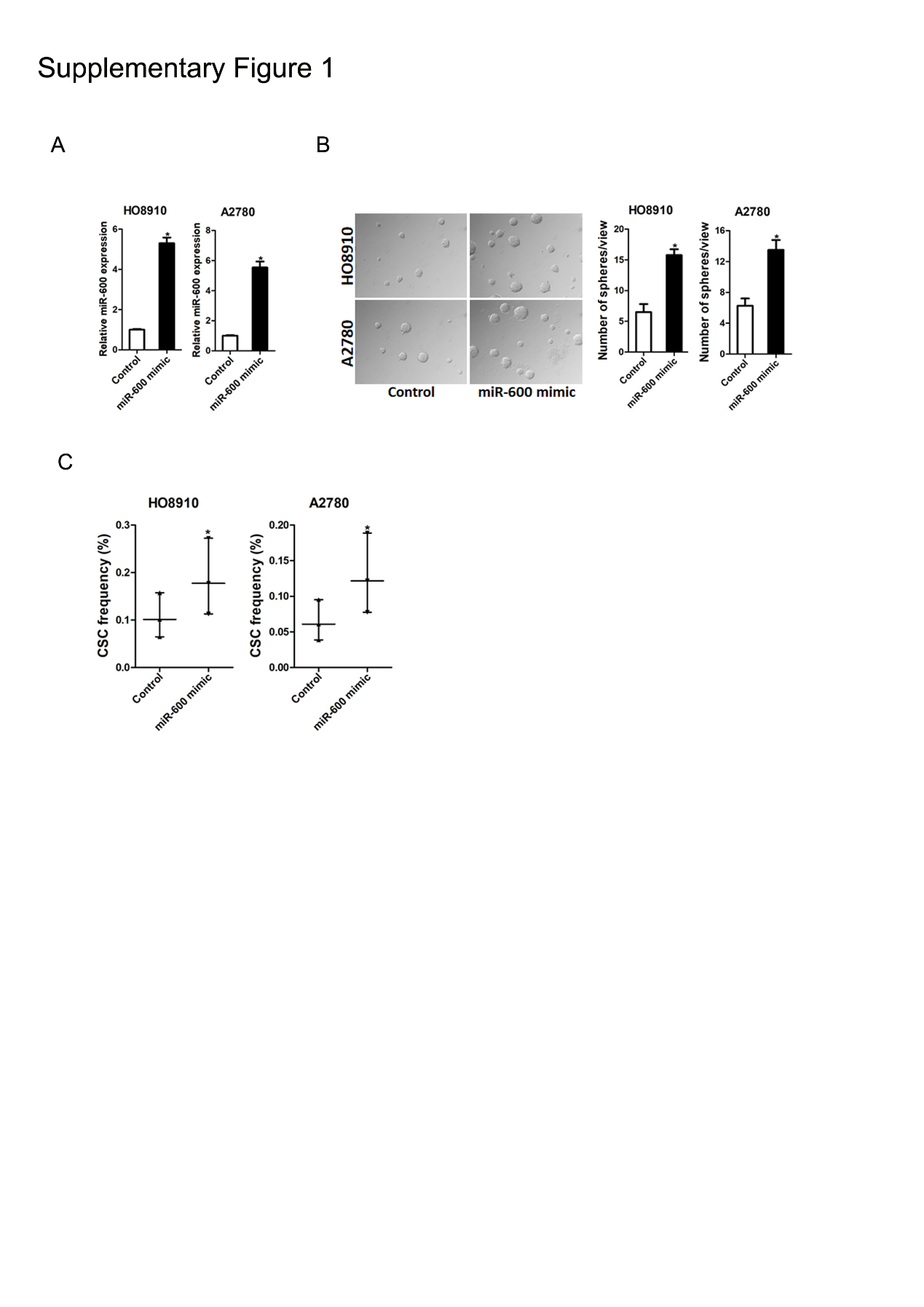


**Supplementary Figure 2**

A. The overexpression effect of miR-600 in HO8910 and A2780 cells was checked by real-time PCR analysis.

B. Representative images of ovarian cancer spheroids generated from miR-600 overexpression ovarian cancer cells and control cells. The number of spheroids was counted and compared.

C. The frequency of ovarian cancer stem cells in miR-600 overexpression ovarian cancer cells and control cells was compared by *in vitro* limiting dilution assay.


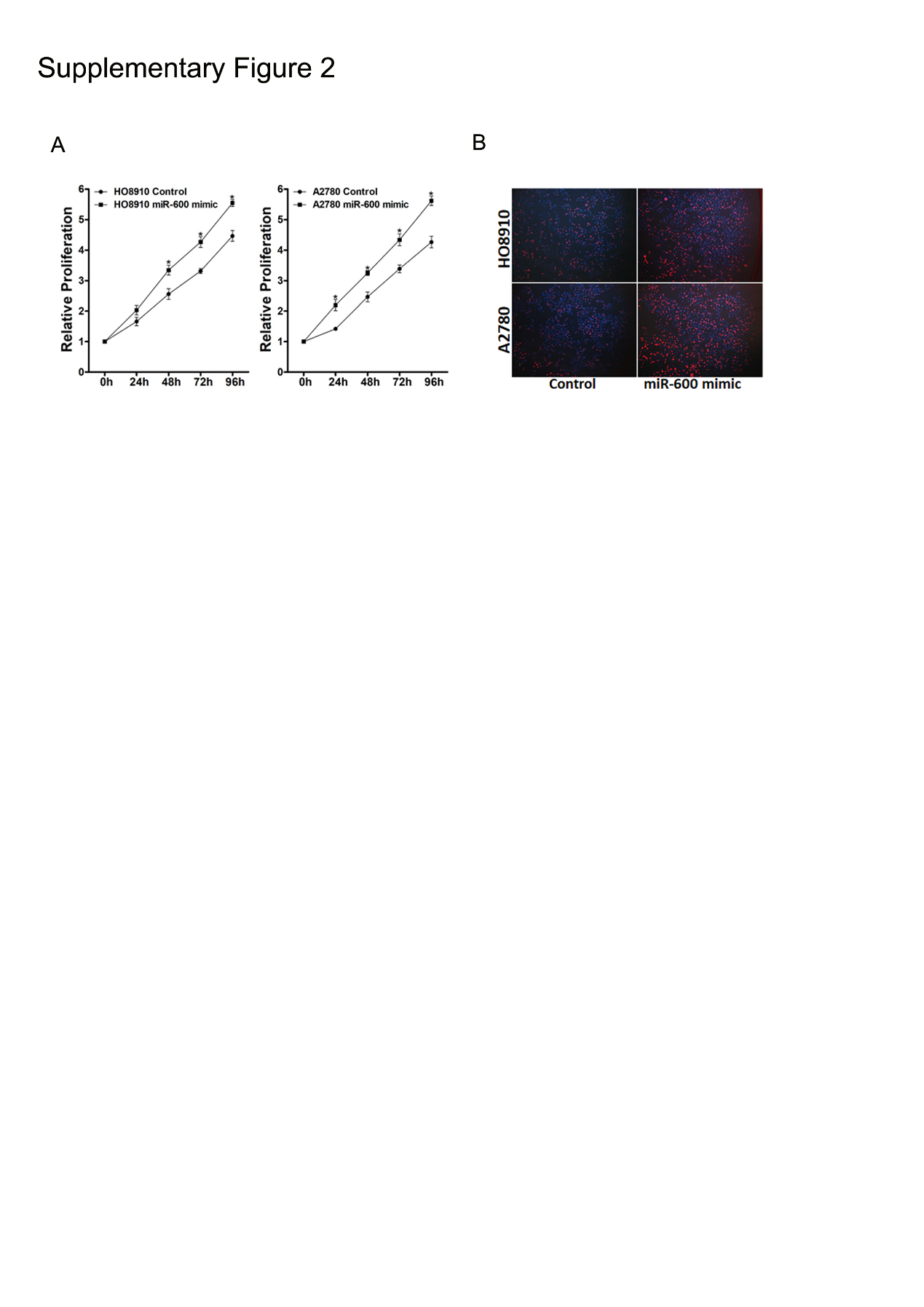


**Supplementary Figure 2**

A. The proliferation curves of miR-600 overexpression ovarian cancer cells and control cells was measured by using CCK-8 assays.

B. The proliferation of miR-600 knockdown overexpression cancer cells and control cells were performed by EdU immunofluorescence staining assay.
